# Testicular tumor arising from an intra-abdominal undescended testis in a 1-year-old child: a case report and literature review

**DOI:** 10.3389/fped.2023.1142157

**Published:** 2023-05-17

**Authors:** Qiongzhang Xia, Tongshuai Kuang, Xiaokun Lin, Hua Zhang

**Affiliations:** ^1^ Department of Pediatric Surgery, The Second Affiliated Hospital and Yuying Children's Hospital of Wenzhou Medical University, Wenzhou, China; ^2^ Wenzhou Key Laboratory of Children Genitourinary Diseases, The Second Affiliated Hospital and Yuying Children's Hospital of Wenzhou Medical University, Wenzhou, China

**Keywords:** testicular tumor, undescended testis, cryptorchidism, teratoma, orchiopexy

## Abstract

**Objectives:**

Testicular tumors in the intra-abdominal undescended testis are rare in children, and their management remains challenging. The aim was to present a case report and review of the literature about diagnosis and treatment of testicular tumors arising from undescended intra-abdominal testis in children.

**Methods:**

In this study, we retrospectively analyzed the clinical records of a 1-year-old male patient admitted to pediatric surgery in March 2022 with a testicular tumor originating in the intra-abdominal undescended testis. Furthermore, medical literature published in English during the last three decades was systematically searched through the databases of Medline, PubMed, and Google Scholar.

**Results:**

The patient underwent laparoscopic orchiopexy and tumor excision. The operation was uneventful, and the patient recovered well without complications. An 8-month follow-up showed no recurrence of the teratoma after postoperative pathology. The literature search resulted in the retrieval of 16 non-duplicate articles, and 16 patients were included in this review. The cases included six cases of left cryptorchidism and 10 cases of right cryptorchidism, with an average age of 15.3 months. The largest transverse diameter of the tumors ranged from 1.8 to 12.5 cm, with an average tumor length of 6.7 cm. All patients underwent surgical treatment, including three cases of laparoscopic orchiectomy, a sole case of a conversion of inguinal incision to laparotomy and orchiectomy, and 12 cases of laparotomy and orchiectomy. Postoperative pathology revealed 12 cases of mature teratoma, two cases of immature teratoma, one case of yolk sac tumor, and a single case of embryonic carcinoma combined with yolk sac tumor. 11 patients were followed up, and one of them recurred.

**Conclusion:**

Abdominal ultrasound (US) or abdominal computer tomography (CT) should be performed in cases of undescended testis suspected to have testicular tumors on clinical findings. The most common type of intra-abdominal testicular tumor is mature teratomas. Early diagnosis and prompt surgical intervention resulted in an excellent outcome.

## Introduction

Undescended testis also called cryptorchidism, is the most common reproductive congenital anomaly in pediatric urology (1). Undescended testis is one of the few known risk factors for testicular tumor (2). The undescended testis increases the risk of testicular tumor 3.7–7.5 times (3). The relative risk to develop testicular tumor for boys who had orchidopexy before puberty ranged from 2.02–2.35, while between 5.06 and 6.24 in those who underwent orchidopexy after puberty (4). Pediatric testicular tumors are rare and represent 1%–2% of all pediatric solid tumors, with an incidence of 0.5–2.0 per 100,000 children (5). Most pre-pubertal testicular tumors are benign compared to post-pubertal tumors, which are 75% malignant (6). The relationship between undescended testis and testicular tumors remains unclear. The causes of children with undescended testicles that have tumors and the timing of tumor development remain unknown. We present a rare testicular tumor from an undescended testis treated with laparoscopic orchiopexy and excision. The patient had cryptorchidism without tumor in the preliminary stages, but a testicular tumor suddenly appeared during follow-up, a finding that has not been documented in the medical literature.

## Materials and methods

### Case presentation

**Figure 1:**
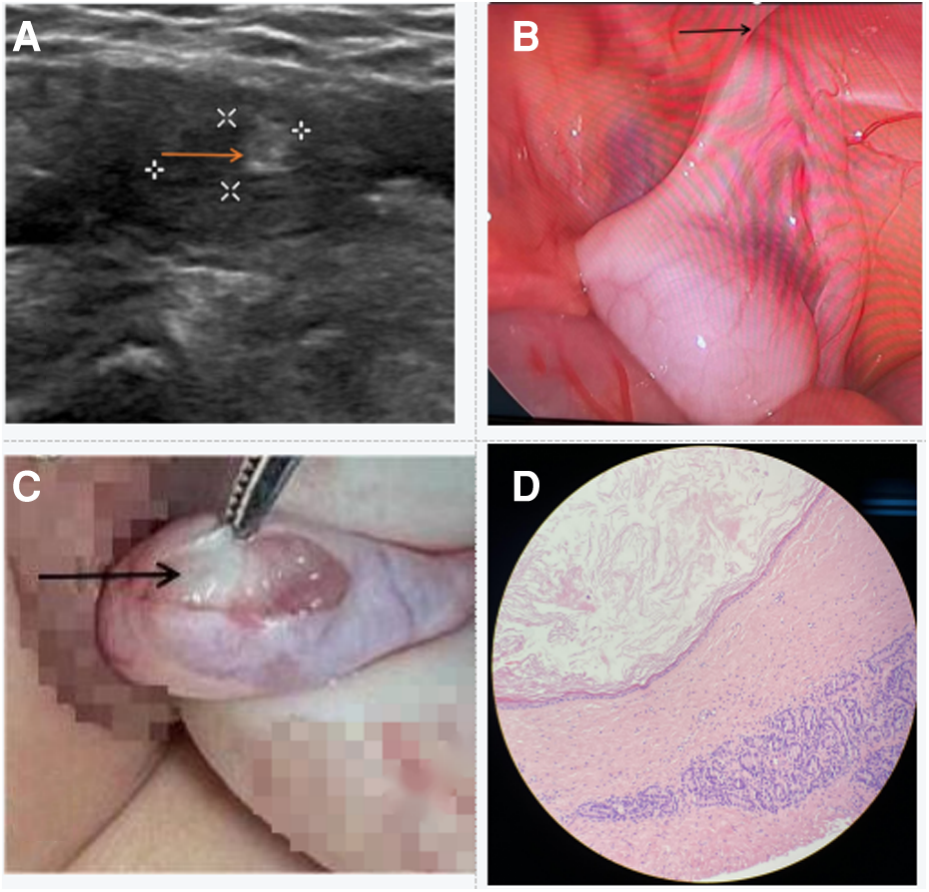
Perioperative data of the patient. (**A**) The US shows hyperechoic changes in undescended testis (see arrow). (**B**) The testicle was located in the abdomen, above the internal ring (see arrow). (**C**) The well-defined, white solid mass was seen in the testicle (see arrow). (**D**) The postoperative pathological examination suggested benign mature teratoma.

A 1-year-old boy, born with a non-palpable undescended testis on the right side, was admitted to our hospital. Four months ago, the first ultrasound (US) examination revealed a nonpalpable undescended testis in the abdomen near the internal ring and no mass was found in the testis. The preoperative ultrasound showed that the testis was still in the abdomen near the internal ring. The US simultaneously showed a hyperechoic mass of approximately 3 mm in the testis (Figure 1A). However, a contrast-enhanced computer tomography (CT) scan showed no mass.

Furthermore, blood routines and coagulation tests were normal. Serum tumor marker analyses also showed no abnormalities: Alpha-fetoprotein (4.62 ng/ml), *β*- hCG (<0.1 mIU/ml). It was necessary for the child to undergo laparoscopic surgery. During the operation, we discovered that the right testicle was within a few centimeters of the internal ring but that there was no visible tumor (Figure 1B). Using a small incision in the middle of the right scrotum, the testis was pulled out through the inguinal canal. A hard mass could be palpated in the upper pole of the testis. The tunica albuginea was dissected by taking appropriate precautions and the testicular tumor were exposed (Figure 1C). We performed testis-sparing surgery. The specimen was sent for rapidly frozen histopathology, and the report suggested a mature teratoma of the testis (Figure 1D). The testis was fixed in the scrotum and the skin was sutured. The patient recovered uneventfully and was discharged on the second postoperative day. After eight months of follow-up, the patient showed no evidence of recurrence and testicular atrophy.

## Literature review

Medical literature published in English during from 1990 to 01-01 to 2022-12-31 was systematically searched through the databases of Medline, PubMed, and Google Scholar. The publication type was limited to article, case report and case series. The main search terms were set as “testicular tumor”, “undescended testis”, and “non-palpable testis”. After excluding patients older than 14 years of age and those children's cases without full-text articles, a total of 16 cases were retrieved from English literatures. Clinical data were counted, including age, clinical manifestations, location, preoperative examination, tumor diameter, surgical method, pathological diagnosis, follow-up time, and recurrence. The clinical characteristics of the patients are shown in Table 1. The details are described in Table 2 (7–22). The onset time was from newborn to five years old, with an average age of 15.3 months. 87.5% of the cases were children under three years; another 12.5% were children between three and five years old. The patients underwent imaging evaluation, including US or CT or magnetic resonance imaging (MRI), during the preoperative period. A total of 13 patients underwent US examination, but only one patient didn't find the testicular tumor. There were multiple variations in the reported US morphologic properties, including a solid, cystic, or mixed mass. The cases consisted of six patients with unilateral left and 10 with unilateral right. Only 1/4 of the cases had obvious clinical symptoms, mainly abdominal pain (three cases) and abdominal mass with pain (one case), and the remaining 3/4 of the cases had no clinical symptoms before imaging examination. The largest transverse diameter of the tumors ranged from 1.8 cm to 12.5 cm, with an average tumor length of 6.7 cm. All patients underwent surgical treatment, including three cases of laparoscopic orchiectomy, a sole case of a conversion of inguinal incision to laparotomy and orchiectomy, and 12 cases of laparotomy and orchiectomy. No case was treated with testis-sparing surgery. Postoperative pathology revealed 12 cases of mature teratoma, two cases of immature teratoma, one case of yolk sac tumor, and a single case of embryonic carcinoma combined with yolk sac tumor. Both immature teratomas were grade 1. One patients relapsed 3 months later, and underwent tumor re-excising, then received chemotherapy with BEP protocol, which included bleomycin, etoposide, and cisplatin. Another patient didn't undergo additional treatment. The patient with embryonic carcinoma combined with yolk sac tumor received one cycle of chemotherapy with bleomycin, etoposide and cisplatinum. The patient with a stage III yolk sac tumor received the first cycle of JEB therapy (which included carboplatin, etoposide and bleomycin) and subsequent PEB therapy consisted of cisplatin, etoposide and bleomycin). Three-month to three-year follow-ups were conducted on 11 cases, and recurrence was noted in only one patient.

**Table 1:** Summary of clinical characteristics based on the literature.

	Number	%
**Age (year)**		
0–3	14	87.5
above 3	2	12.5
**Location**		
Left	6	37.5
Right	10	62.5
**Clinical manifestation**		
Asymptomatic	12	75
Abdominal pain	3	18.75
mass and abdominal pain	1	6.25
**Diameter of tumor (cm)**		
0–4	4	25
4–8	5	31.25
8–13	6	37.5
Not mentioned	1	6.25
**Operation method**		
Laparotomy	12	75
Inguinal and laparotomy	1	6.25
Laparoscope	3	18.75
**Pathologic diagnosis**		
Mature teratoma	12	75
Immature teratoma	2	12.5
Yolk sac tumor	1	6.25
Embryonic carcinoma with yolk sac tumor	1	6.25

**Table 2:** Clinical features of 16 patients based on the literature.

Literature resources	Age of children	Operation Position	Preopreative examination	Tumor diameter (cm)	AFP	*β*-HCG (MIU/ml)	Surgical method	Pathological diagnosis	Recurrence
Shih (7)	6 days	Right	US&CT	5	911U/l	Normal	Laparotomy and orchiectomy	MT	No
Mboyo (8)	13 days	Left	US	10	7,980 mg / ml	/	Laparotomy and orchiectomy	MT	No
Janda (9)	19 days	Left	US	1.8	/	/	Laparotomy and orchiectomy	MT	/
Siu (10)	1 months	Right	US	2.7	Normal	Normal	Laparoscopic orchiectomy	MT	/
Tanaka (11)	2 months	Left	US&CT	12.5	Normal	/	Laparotomy and orchiectomy	MT	No
Hasegawa (12)	3 months	Right	US&CT	11	Normal	Normal	Laparotomy and orchiectomy	IMT	YES
Azumagawa (13)	4 months	Left	US&CT&MRI	4	150 ng/ml	Normal	Laparotomy and orchiectomy	MT	No
Schwabe (14)	5 months	Right	US	7	Normal	Normal	Laparotomy and orchiectomy	IMT	No
Yam (15)	7 months	Right	US&CT	8.6	29 ng/ml	/	Laparotomy and orchiectomy	MT	Loss to follow-up
Brown (16)	7 months	Right	US	5.5	Normal	Normal	Laparotomy and orchiectomy	MT	No
Mukai (17)	23 months	Right	US	3.2	Normal	Normal	Laparoscopic orchiectomy	MT	/
Nakamoto (18)	2 years	Right	/	3.8	/	/	Inguinal&laparotomy and orchiectomy	MT	No
Hirayama (19)	2 years	Left	CT	10	36,528 ng/ml	/	Laparotomy and orchiectomy	YST	No
Deb (20)	3 years	Left	US&CT	10	250 ng/ml	1.47	Laparotomy and orchiectomy	EC&YST	Loss to follow-up
Agarwal (21)	4 years	Right	US	/	/	/	Laparoscopic orchiectomy	MT	No
Doi (22)	5 years	Right	CT	4.8	Normal	Normal	Laparotomy and orchiectomy	MT	No

US, ultrasound; CT, computer tomography; MRI, magnetic resonance imaging; MT, mature teratoma; IMT, Immature teratoma; YST, yolk sac tumor; EC&YST, embryonic carcinoma with yolk sac tumor, /:missing.

## Discussion

Studies have found that 20% of undescended testis were non-palpable, and 25%–40% were non-palpable intraabdominal testes (23, 24).Undescended testis had an incidence of 1%–3% in full-term male newborns and about 0.8% at one year of life. It is estimated that undescended testis cause around 10% of all testicular tumors, with the risk being higher for testicles in the abdomen (25). However, the molecular mechanism behind the development of malignancy and failed testicular descent remains unknown. Testicular tumors in children are rare in clinics, the most common of which are teratomas found in pre-pubertal children (26). However, intra-abdominal testicular tumor originating in the abdominal cavity is even rarer. Based on the literature review, non-palpable intraabdominal testis with testicular tumor are more commonly observed in children under two years old. When the intra-abdominal testicular tumor is located above the inner ring, it may prevent the testicle from descending properly into the scrotum (17).

A typical clinical presentation is absent in patients with undescended testis or testicular tumors. Most patients were hospitalized for cryptorchidism, and testicular tumors were discovered unexpectedly during the preoperative ultrasound. Clinical symptoms are less apparent when the patient is young, even asymptomatic. This may be due to the children's inability to express symptoms and the fact that symptoms are ignored, leading to late detection. Cryptorchidism with testicular tumors has only been diagnosed in a few patients due to abdominal mass or abdominal pain. Testicular tumors in the abdominal cavity are difficult to detect without imaging examination. The abdominal US is sensitive to testicular tumors, with hyperechoic changes often indicating tumors. An abdominal CT examination plays a vital role in confirming an intraperitoneal undescended testis with testicular tumor (27).

The most common use of this procedure is a follow-up examination after the tumor is discovered to clarify diagnoses and determine whether it has metastasized. However, some testicular tumors were too small to be detected on abdominal CT. In the reported case, only an undescended testis was found in the abdominal cavity at diagnosis and the abdominal US did not indicate a tumor. On abdominal CT, however, there was no sign of a testicular tumor four months later, even though the abdominal US showed a testicular tumor in the undescended testis. The preoperative diagnostic work-up includes imaging data and blood samples for tumor markers such as AFP, *β*-HCG, and CA-125 (28). An auxiliary examination was used to identify the type of tumor before surgery, but postoperative pathology determined the final diagnosis. Testicular tumors with undescended testis have many histologies, including benign types, such as teratomas, and some malignant types, such as yolk sac tumors and seminomas. There were 17 children in review of literature, 15 with teratomas (13 mature and two immature), one child with a yolk sac tumor, and a child with an embryonic carcinoma combined with a yolk sac tumor.

Surgical resection is critical for the treatment of patients with undescended testis and testicular tumors. A frozen-section biopsy performed during surgery may determine the best surgical procedure. The preservation of gonad function and mass resection are crucial for children whose tumors are benign. Testis-sparing surgery is applied to benign tumors and a microscopically negative resection margin (R0) must be guaranteed (29). Radical surgical excision is recommended if the tumor is malignant. However, there is still controversy concerning the use of retroperitoneal lymph node dissection (RPLND) (30). RPLND are often used in children with elevated or rising AFP levels and/or retroperitoneal lymphadenopathy (31). Following surgery, the patient received chemotherapy or radiotherapy, as necessary. The surgical approach is also determined by the size of the intraperitoneal testicular tumor. There are few reports in the literature on surgical methods, and most focus on open surgical methods. Laparotomy is recommended if the tumor size is larger than 3 cm, or the nature of the tumor cannot be determined before the operation. Based on the literature review, 12 cases (75%) performed laparotomy. Our experience suggests that laparoscopic surgery might be viable if the tumor is small, especially smaller than 1 cm. Following laparoscopic orchiopexy and tumor excision, there was no recurrence and no testicular atrophy during the follow-up period.

In conclusion, abdominal US or abdominal CT should be performed in cases of undescended testis suspected to have testicular tumors on clinical findings. The most common type of intra-abdominal testicular tumor is mature teratomas. Early diagnosis and prompt surgical intervention resulted in an excellent outcome.

## Data Availability

The raw data supporting the conclusions of this article will be made available by the authors, without undue reservation.
